# The number of undocumented immigrants in the United States: Estimates based on demographic modeling with data from 1990 to 2016

**DOI:** 10.1371/journal.pone.0201193

**Published:** 2018-09-21

**Authors:** Mohammad M. Fazel-Zarandi, Jonathan S. Feinstein, Edward H. Kaplan

**Affiliations:** 1 Yale School of Management, Yale University, New Haven, CT, United States of America; 2 Sloan School of Management, Massachusetts Institute of Technology, Cambridge, MA, United States of America; 3 Yale School of Public Health, Yale University, New Haven, CT, United States of America; 4 Yale School of Engineering and Applied Science, Yale University, New Haven, CT, United States of America; Stanford University, UNITED STATES

## Abstract

We apply standard demographic principles of inflows and outflows to estimate the number of undocumented immigrants in the United States, using the best available data, including some that have only recently become available. Our analysis covers the years 1990 to 2016. We develop an estimate of the number of undocumented immigrants based on parameter values that tend to underestimate undocumented immigrant inflows and overstate outflows; we also show the probability distribution for the number of undocumented immigrants based on simulating our model over parameter value ranges. Our conservative estimate is 16.7 million for 2016, nearly fifty percent higher than the most prominent current estimate of 11.3 million, which is based on survey data and thus different sources and methods. The mean estimate based on our simulation analysis is 22.1 million, essentially double the current widely accepted estimate. Our model predicts a similar trajectory of growth in the number of undocumented immigrants over the years of our analysis, but at a higher level. While our analysis delivers different results, we note that it is based on many assumptions. The most critical of these concern border apprehension rates and voluntary emigration rates of undocumented immigrants in the U.S. These rates are uncertain, especially in the 1990’s and early 2000’s, which is when—both based on our modeling and the very different survey data approach—the number of undocumented immigrants increases most significantly. Our results, while based on a number of assumptions and uncertainties, could help frame debates about policies whose consequences depend on the number of undocumented immigrants in the United States.

## Introduction

Immigration policy remains a hotly debated issue in the United States, with perhaps no aspect more controversial than how to address undocumented immigrants who do not have legal status. Policy debates about the amount of resources to devote to this issue, and the merits of alternative policies, including deportation, amnesty, and border control, depend critically on estimates of the number of undocumented immigrants in the U.S., which sets the scale of the issue. The most widely accepted estimate of this number currently is approximately 11.3 million [1, 2]. This estimate is based on variants of the residual method [2–4]. In this method, the size of the unauthorized immigrant population residing in the United States is set equal to the estimate of the total foreign-born population minus the legally resident foreign-born population. The total foreign-born population estimate is derived from surveys that ask respondents whether they were born outside of the United States (and whether they are American citizens), specifically either the American Community Survey or the Current Population Survey. The legally resident foreign-born population is estimated using administrative data on legal admissions.

An alternative approach to estimating the size of the undocumented population follows directly from basic demographic principles. Starting from a known population size at a given date, the population size at a future date equals the starting value plus the cumulative inflows minus the cumulative outflows. We employ this approach to estimate the number of undocumented immigrants in the U.S. for each year from 1990 to 2016, using the best available data and parameter values from the academic literature and government sources. Some of the information we use has been collected and made available only recently, so our approach is timely.

Our analysis has two main outputs. First, we generate what we call our conservative estimate, using parameter values that intentionally underestimate population inflows and overestimate population outflows, leading to estimates that will tend to underestimate the number of undocumented immigrants. Our conservative estimate for 2016 is 16.7 million, well above the estimate that is most widely accepted at present, which is for 2015 but should be comparable. Our model as well as most work in the literature indicates that the population size has been relatively stable since 2008; thus 2015 and 2016 are quite comparable. For our second step, recognizing that there is significant uncertainty about population flows, we simulate our model over a wide range of values for key parameters. These parameter values range from very conservative estimates to standard values in the literature. We sample values for each key parameter from uniform distributions over the ranges we establish. In our simulations, we also include Poisson population uncertainty conditional on parameter values, thus addressing the inherent variability in population flows. Our simulation results produce probability distributions over the number of undocumented immigrants for each year from 1990 to 2016. The results demonstrate that our conservative estimate falls towards the bottom of the probability distribution, at approximately the 2.5th percentile. The mean of the 2016 distribution is 22.1 million, which we take as the best overall estimate of the number of undocumented immigrants based on our modeling approach and current data. We also show the variability in our model based on the simulations for each year from 1990 through 2016.

## Methods

The model works as follows (mathematical formulation, parameter values, and data sources underlying this model are detailed in the Supporting Information). For our conservative estimate we begin with a starting 1990 population of 3.5 million undocumented immigrants, in agreement with the standard estimate [1]. The estimate of 3.5 million undocumented immigrants in 1990 is based on applying the residual method (using the 1980 and 1990 censuses), described previously, which we argue systematically underestimates the population. Thus in assuming an initial population of 3.5 million, and centering our simulations around this value, we are almost certainly underestimating the size of the undocumented immigrant population at this date. In the simulations we assume that the starting population is drawn from a Poisson distribution with a mean of 3.5 million. It then follows that the population size at a future date equals the starting value plus the cumulative inflows minus the cumulative outflows.

### Population inflows

Population inflows are decomposed into two streams: (I) undocumented immigrants who initially entered the country legally but have overstayed their visas; and (II) immigrants who have illegally crossed the border without being apprehended. We describe our approach for each source, explain the basis for our assumptions and why they are conservative, and list parameter ranges for the simulation.

**(I) Visa overstays** are estimated using Department of Homeland Security (DHS) data for 2016, the first year for which visa overstays were comprehensively measured [5]. To apply this data in our context we also gather data for non-immigrant visas issued for all years from 1990 [6]. For our conservative estimate we assume that for each year the rate of overstays was equal to the 2016 rate. Calibration of our model shows that this assumption is in fact quite conservative. In particular, approximately 41% of undocumented immigrants based on the current survey data approach are visa overstayers [7], which translates to a visa overstay population of 4.6 million in 2015. Our model however predicts the number of overstayers to be less than this (even though our overall estimate of the number of undocumented immigrants is higher). That is, in our model most undocumented immigrants are not overstayers, and the model produces an estimate of the number of overstayers below the estimate produced in the conventional approach based on survey data. We compute that we would need to set the visa overstay rate above the DHS 2016 rate, specifically 1.1 times that rate, for our conservative estimate to generate as many overstayers as the 4.6 million in the 11.3 million estimate. Since many overstayers leave or adjust their status within a few months of their visa expiration date, we make a further conservative adjustment and count as overstayers only those individuals who have overstayed more than 1 year. For the simulation, we set the visa overstay rate equal to the 2016 rate multiplied by a uniform draw from the range [0.5,1.5]; consistent with the discussion above, this is a relatively conservative range.

**(II) Illegal Border Crossers:** We estimate illegal border crossers through application of the standard repeated trials (capture-recapture) model [8–10]. The model requires as inputs statistics on the total number of border apprehensions, the number of individuals apprehended more than once in a year (recidivist apprehensions), and estimates of the deterrence rate—the fraction of individuals who give up after being apprehended and do not attempt another crossing. Given these inputs, the repeated trials model generates estimates of: (i) the apprehension rate—the probability an individual is caught trying to cross the border; and (ii) the total number of individuals who are not apprehended (they may be caught one or more times but cross successfully on a later attempt) and enter the interior of the country illegally—the number of illegal border crossers in a year. We discuss data sources and potential weaknesses of this approach here; more information and mathematical details are provided in the Supporting Information.

DHS [10, 11] provide figures for the total number of border apprehensions for every year in our timespan. They also provide information on the number of recidivist apprehensions and estimates of the deterrence rate for every year from 2005. Based on these figures and estimates they provide an estimate of the apprehension rate for each year from 2005 to 2015. Their estimate is 35% for 2005 and increases steadily, to above 50% by the end of the sample period. From their estimates we are able to derive directly estimates of the number of illegal border crossers for each of these years. For earlier years (1990 to 2004) we must make further assumptions. Our assumptions are about the apprehension and deterrence rates, since these have been addressed in the literature; in turn we are able to generate estimates of the number of illegal border crossers in earlier years based on these assumptions (see the Supporting Information for analytic details).

Most experts agree that the apprehension rate was significantly lower in earlier years [12, 13]. A recent study [12] using data from the Mexican Migration Project estimates this rate for every year from 1990 to 2010; estimates in the 1990’s begin from the low twenties and range upwards to approximately 30%. A second study estimates the rate for 2003 at around 20% [13]. Given these estimates, and the general view that apprehension rates have risen, for our conservative estimate we assume that the apprehension rate in years 1990-2004 was equal to the average rate in years 2005-10 or 39%; this is well above the rates discussed in the literature for earlier years and thus tends to reduce our estimate of the number of undocumented immigrants since it implies a larger fraction are apprehended at the border. For our simulation we assume a uniform distribution over the range [0.25,0.40] for the earlier years, still above the average rates in the literature for these years.

Additional facts support the view that the apprehension rate has increased in recent years. The number of border agents has increased dramatically over the timespan of our analysis [14], and the number of hours spent by border agents patrolling the immediate border area has increased by more than 300% between 1992- 2004 [15]. Further, new infrastructure (e.g., fences) and technologies (e.g., night vision equipment, sensors, and video imaging systems) were also introduced during this period [15]. Thus the apprehension rate we use for earlier years almost certainly overstates the actual apprehension rate and therefore underestimates the number of successful crossings. However, we note that these additional border resources may have been concentrated in certain locations and it remains a possibility that apprehension rates were higher in earlier years. We note finally that in using data only on Southern Border crossings we again are conservative in our approach, not accounting for illegal crossings along other borders.

Notwithstanding our view that we make conservative choices in setting up our model and parameter values, we acknowledge that border apprehension rates for the 1990’s are not based on as well-developed data sources as estimates for more recent years. Thus it remains a possibility that these rates are higher than we believe. One aspect of this uncertainty concerns deterrence. When deterrence is higher border crossings will fall. Most researchers believe deterrence has increased in recent years [8, 12]. We note that reference [12] estimates that the probability of eventual entry after multiple attempts on a single trip in the 1990s is close to one, indicating almost no deterrence in the earlier period. One piece of evidence in support of this is data on the voluntary return rate, which refers to the percentage of individuals apprehended at the border who are released back to their home country without going through formal removal proceedings and not being subjected to further penalties. Voluntary returns are thus not “punished” and thus are less likely to be deterred from trying to cross the border in the future, compared with individuals who are subjected to stronger penalties. The voluntary return rate has fallen in recent years, from 98% between 2000 and 2004 to 84% between 2005 and 2010. Thus, at least based on this measure deterrence efforts have increased. However, this does not conclusively demonstrate that deterrence was lower in earlier years and it remains a possibility that it was higher, which would tend to reduce our estimates of the number of undocumented immigrants. In conclusion we note that although there is much uncertainty about the border apprehension rate, it would have to be very high, above 60% for earlier years, in order to generate estimates of the 2015 population of undocumented immigrants in the range of the current widely accepted estimate of just over 11 million (this is based on analyzing our model using the conservative estimate values for all other parameters). This seems implausible based on our reading of the literature.

### Population outflows

Population outflows are broken into four categories: (I) voluntary emigration; (II) mortality; (III) deportation; and (IV) change of status from unauthorized to lawful.

**(I) Voluntary emigration rates** are the largest source of outflow and the most uncertain based on limited data availability. It is well accepted that voluntary emigration rates decline sharply with time spent in the country [16]; thus we employ separate emigration rates for those who have spent one year or less in the U.S., 2-10 years, or longer. We use the following values for our conservative estimate. First, for those who have spent one year or less we assume a voluntary emigration rate of 40%. This estimate is based on data for the first-year visa overstay exit rate (the fraction of overstayers who left the country within one year from the day their visa expired) for 2016 [17], which is in the lower thirty percent range (the rate for 2015 is similar). We note that the rate for visa overstayers is very likely a substantial overestimate for illegal border crossers, who are widely viewed as having a lower likelihood of exiting in the first year, especially in more recent years [12]. The 40% first-year emigration rate that we assume is well above the standard values in the literature [4, 12, 16, 18], which range from 1% to 25%. Hence this assumption contributes to making our estimate of the number of undocumented immigrants in the country a conservative one. For years 2-10 we assume a rate of 4% per year. This is the upper bound among estimates in the literature, which lie between 0.01 to 0.04 [4, 16, 18]. Lastly, for years 10 and above, published estimates of the emigration rate typically fall around 1%; we set this rate to 1% per year in line with these estimates. Note that given the extremely high 40% emigration rate that we assume for those who have only been in the country for one year or less, overall annual emigration rates in our model simulation are significantly higher than those found in the literature or government sources. To further enhance the conservatism of our model, we assume that all undocumented immigrants present at the beginning of 1990 have been here for only one year.

For our simulation analysis we divide first-year voluntary emigration into two categories, visa overstayers and illegal border crossers. For visa overstayers we assume the first-year rate falls in the range [.25,.50] (uniform) for each year; based on the discussion in the preceding paragraph and literature cited there, this is a relatively conservative range with midpoint 37.5% above nearly all accepted estimates. For illegal border crossers there is data indicating that first-year voluntary emigration rates vary across cohorts [12] (we are not aware of such data for visa overstayers). To incorporate this, we assume that a voluntary emigration rate is drawn for each cohort year from a uniform distribution that is specific to that cohort’s year of initial entry; the lower bound of this range is set by the numbers in [12] and the upper bound is set at 0.50. Again our assumptions here are conservative, since we use an accepted value in the literature as our lower bound and allow emigration rates to range to very high values. For years 2-10 and 10 and above we use the same distributions for overstayers and illegal border crossers. For years 2-10 we draw a value from the range [.01, .05], for which the mean value of 3% is relatively high and thus conservative; and for years 10 and above we draw a value from the range [.005,.02], thus centered slightly above the standard value in the literature. We note that the first-year rate is the most critical for our analysis.

An important issue is circular flow of migrants, which refers to individuals who enter the country, then exit temporarily and re-enter a short time later. There is limited numerical data for circular flow rates. However, it is logical and recognized in the literature [12] that when border apprehension rates are higher circular flow rates for border crossers tend to diminish: Given it will be more difficult to re-enter the country successfully later, illegal border crossers in the country will tend not to leave for temporary reasons. Thus this issue is important for illegal border crossers (but not likely to be as relevant for visa overstayers). Thus in our simulation we impose a negative correlation between the first-year emigration rate and the border apprehension rate for illegal border crossers; based on our own analysis for annual data from the best recent study [12] we use a correlation of -0.5 (see the Supporting Information for details). We note that this correlation does not substantially change the range or mean of our simulation results, but does reduce the variance.

**(II) The mortality rate** applied is the age-adjusted mortality rate reported by the Centers for Disease Control and Prevention [19]. For our conservative estimate we set this value at 0.7 percent, and for the simulation we draw a value from the range [0.5,1.0] percent. We view these values as conservative. Experts in the field argue that this rate overestimates mortality among undocumented immigrants [4]. To further check that our mortality rate assumptions are an overestimate and thus contribute to making our overall estimate of the number of undocumented immigrants conservative, we combined the age, gender, and country of birth distributions of undocumented immigrants reported in [2, 20] with CDC mortality rates [19]. The resulting mortality rate is much lower than the mortality rate we assume (see the Supporting Information for details). We note that the mortality rate is low relative to the voluntary emigration rate, and thus a less important parameter for the calculation we make.

Lastly, **(III)** the annual number of deportations is taken directly from DHS annual statistics [11, 21] for each year. **(IV)** The number of undocumented immigrants who change to legal status in each year is also taken directly from published data [4, 11]. We include the number of deferred action for childhood arrivals (DACA) recipients as population outflows even though such individuals remain technically undocumented, which again serves to underestimate the size of the population.

### Simulation methodology

Our simulation is designed to evaluate the range of outcomes the model produces, thus taking into account important sources of variability. There are two main sources of uncertainty: parameter uncertainty, and inherent population variability conditional upon a set of parameter values. We take both sources into account, but note that the first source is the main factor contributing to the variability of the population distribution in the model.

We address parameter uncertainty by establishing ranges for key parameters. As documented above, these key parameters are (i) the visa overstay rate; (ii) the border apprehension rate for individuals attempting to cross the border illegally; (iii) the voluntary emigration rate, which is set separately for illegal border crossers and visa overstayers for the first year and then jointly for years 2-10 and years 10 and above, and for which we establish a cohort-specific range for each annual cohort for the first-year rate for illegal border crossers; and (iv) the mortality rate. For each parameter, we establish a uniform distribution over the set range (and impose a negative correlation between the border apprehension rate and first-year voluntary emigration rate for illegal border crossers). Then, in each simulation run we sample a value for each parameter from its underlying distribution. All of the ranges for the parameter distributions have been specified in the preceding sections. We also sample a value for the initial population of undocumented immigrants in 1990 from a Poisson distribution with a mean of 3.5 million, the most widely accepted estimate of the population of undocumented immigrants as of that date. See the Supporting Information for further details.

To model inherent population uncertainty given a set of parameter values, we impose a Poisson structure on our model. Specifically, the population in a particular year, conditional on a set of parameter values, is represented as the sum of all individuals who have entered the country in previous years and have remained in the country from their year of arrival until the particular year in question. The number of entries (in Poisson terminology, arrivals) in any year is drawn from a Poisson distribution with mean dependent upon the underlying parameter values governing apprehension probabilities and visa overstays for that year, while the probability that a new immigrant remains in the country from entry until the particular year in question is determined based on the parameters governing voluntary emigration, mortality, deportation and change-of-status rates. It follows (see the Supporting Information for mathematical details) that the number of individuals who enter the country in any given year and are still in the country at some future date will also follow a Poisson distribution. Further, the number of individuals who enter in any given year and remain in the country at a future time can be considered to be statistically independent given the underlying parameter values (see the Supporting Information for details). Thus, the population of undocumented immigrants in a particular year, which is the sum of those who have entered in past years and are still in the country in the particular year in question, also follows a Poisson distribution, for the sum of independent Poisson random variables is itself Poisson distributed.

We ran 1,000,000 trials simulating the model. For each trial we recorded the total number of undocumented immigrants predicted to be in the U.S. in each year from 1990 through 2016 for that trial.

Following suggestions made by the Academic Editor based on comments made by a reviewer, we performed an additional set of simulations making even more conservative assumptions about net inflows over the period 1990-98. This is the period for which there is significant uncertainty about net inflows of undocumented immigrants. Specifically, we calibrated the model such that the net inflows are half a million per year over this period (in line with the residual method’s estimates during this period) and computed the pooled number of undocumented immigrants at the end of 1998 based on this approach. We then simulated our model forward from that point using the same framework described above.

## Results

**Fig 1:**
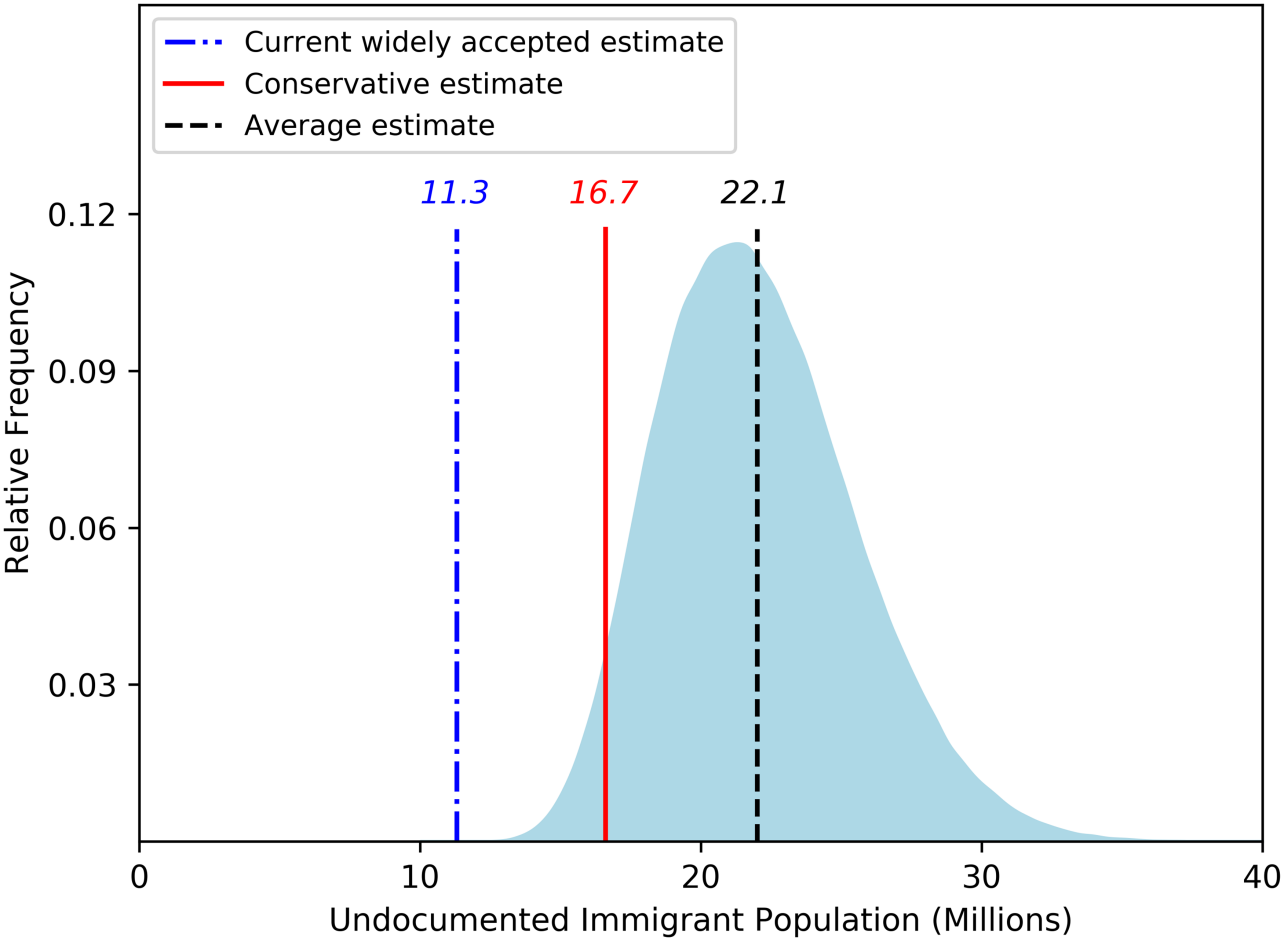
Relative frequency probability function for the number of undocumented immigrants in the U.S.

Fig 1 depicts our results for year 2016, the most recent year for which we are able to produce an estimate. The graph depicts the relative frequency of the number of undocumented immigrants in the U.S.; it is a smoothed version of the histogram we generate based on simulating our model 1,000,000 times. The figure also shows our conservative estimate of 16.7 million in Red, and the most widely accepted estimate heretofore of 11.3 million in Blue on the far left. We note that this last estimate is for 2015, but should be comparable since both the estimates based on the survey approach and our modeling approach indicate that the number of undocumented immigrants has remained relatively constant in recent years. Finally, the mean estimate of 22.1 million is shown in black in the center of the distribution. It is clear from the Figure that based on the data we use, our assumptions, and our demographic model, the currently accepted estimate falls outside the range of likely values. And our conservative estimate is indeed conservative based on our modeling approach and parameter ranges, lying at approximately the 2.5th percentile of the probability distribution.

**Fig 2:**
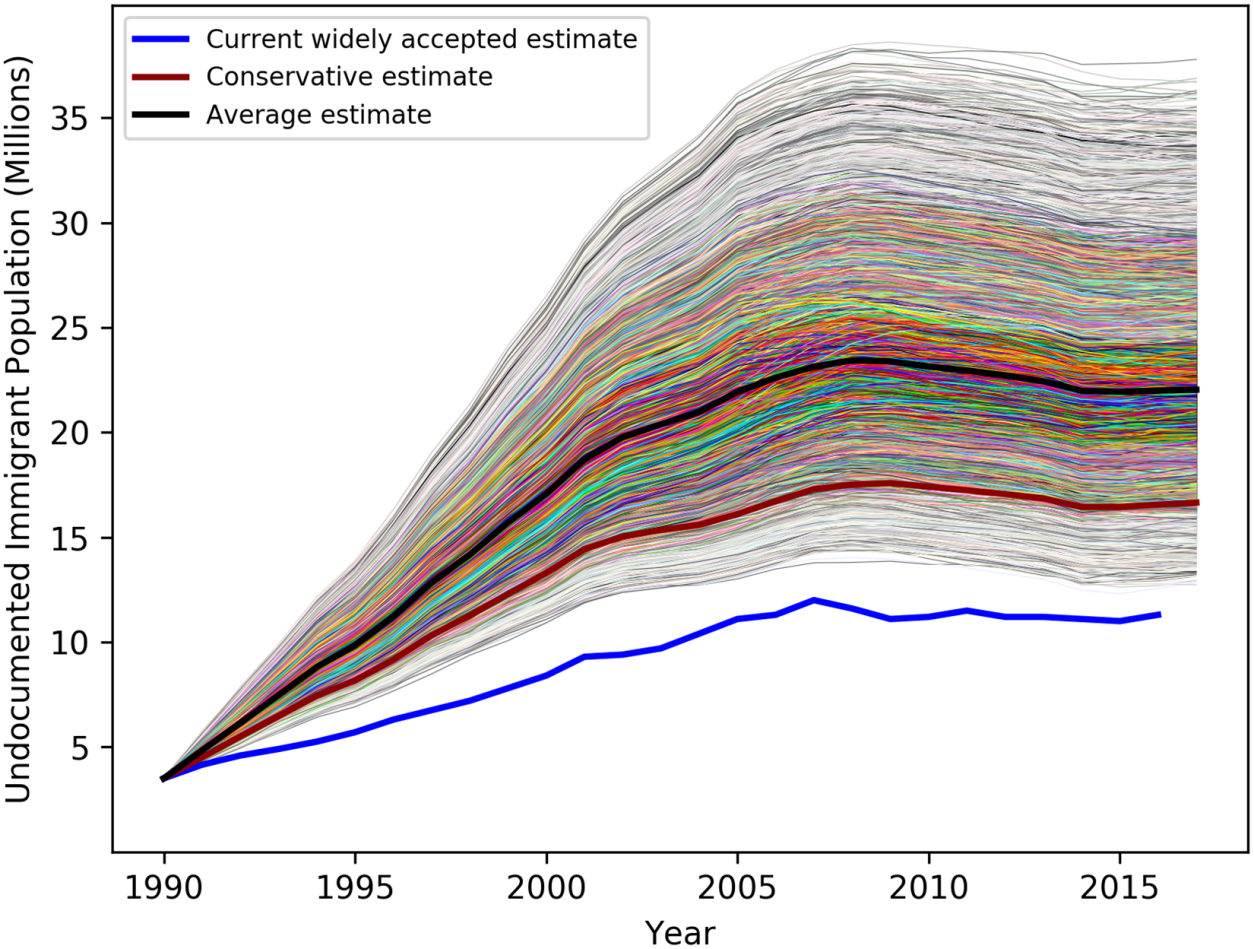
The current widely accepted estimate of the number of undocumented immigrants in the U.S. (in Blue); together with our conservative estimate (in Red); and the mean value we estimate for each year (in Black).

Fig 2 displays our simulation results for each year from 1990 through 2016. Our conservative estimate of the number of undocumented immigrants for each year is shown in Red, the most widely accepted estimate (through 2015) is shown in Blue, and the mean value we estimate for each year is shown in Black. The results show that our model estimates follow a similar shaped trajectory as the widely accepted current estimates do, but grow faster and are well above those estimates for every year.

The results of our analysis are clear: The number of undocumented immigrants in the United States is estimated to be substantially larger than has been appreciated at least in widely accepted previous estimates. Even an estimate based on what we view as conservative assumptions, in some cases unrealistically so, generates an estimate of 16.7 million, well above the conventional estimate of 11.3 million. The mean of our simulations, which range over more standard but still conservative parameter values, is 22.1 million, essentially twice the current widely accepted estimate; the ninety-five percent probability interval is [16.2,29.5].

Even for the scenario presuming net inflows of 0.5 million per year for 1990-98 our results still exceed the current estimates substantially. The mean estimate is 17.0 million with a 95% probability interval of 13.5 million to 21.1 million. The conservative estimate for this scenario is 14.0 million, still significantly above the widely accepted estimate of 11.3 million.

## Discussion

It is currently fairly widely accepted that there are approximately 11 million undocumented immigrants in the United States. This estimate, derived from population surveys and legal immigration records, has formed the backdrop for the immigration policy debate in the United States. Using a different approach grounded in operational data, and demographic and mathematical modeling, we have arrived at higher estimates of the undocumented immigrant population.

A possible explanation for the discrepancy in these results is that the survey-based approach taken in [2–4] must surmount two challenges. First, it requires reaching a representative sample of all those born outside of the United States. Second, it requires accurate responses from survey respondents when asked where they were born, and whether they are American citizens. It is plausible that undocumented immigrants are more difficult to locate (and survey) than other foreign-born residents of the United States, and if contacted, undocumented immigrants might misreport their country of origin, citizenship, and/or number of household residents fearing the possible consequences of revealing their true status. Any of these circumstances would lead to underestimating the true number of undocumented immigrants.

Our approach, summarized above and detailed in the Supporting Information, is grounded in fundamental principles of demographic flows. The size of any population can be represented as its initial value plus cumulative inflows minus cumulative outflows. We have specialized this approach to the number of undocumented immigrants in the United States, and have drawn upon previously unavailable data. From border apprehensions and visa overstays, it is possible to infer the number of new undocumented arrivals by reversing the flow: how many new arrivals are necessary in order to see the number of apprehensions and visa overstayers observed? Similarly, consideration of deportations, voluntary emigration, mortality and change-of-status enables one to infer the duration of stay in the country from the time of arrival. Together, this logic enables reconstructing the arrival and departure processes governing population inflows and outflows that result in the population of undocumented immigrants in the country.

In developing estimates we have attempted to utilize parameter values that understate inflows and overstate outflows. Our results are most sensitive to the assumptions we make about the probability of border apprehension and the voluntary emigration rates of undocumented immigrants leaving the United States. Further research could explore in greater detail the impact of assumptions about these parameters on estimates of the number of undocumented immigrants. To explore the uncertainty of our estimates we have conducted extension simulations over parameters, simulating 1 million different population trajectories; further research could widen the ranges of parameters and consider additional parameter uncertainty. Further research could also analyze inflows and outflows based on country of origin.

Our results lead us to the conclusion that the widely accepted estimate of 11.3 million undocumented immigrants in the United States is too small. Our model estimates indicate that the true number is likely to be larger, with an estimated ninety-five percent probability interval ranging from 16.2 to 29.5 million undocumented immigrants.

## Supporting information

S1 File Supporting material. Contains the mathematical model, parameter values, and data sources underlying the model.
(PDF)

S2 File Excel file. The spreadsheet used to calculate the conservative estimate.
(XLSX)
