# Supplementary material for: The number of undocumented immigrants in the United States: Estimates based on demographic modeling with data from 1990 to 2016
Source: PLoS One. 2018 Sep 21;13(9):e0201193. doi: 10.1371/journal.pone.0201193 (PMC6150478; doi:10.1371/journal.pone.0201193)
Supplement: S1 File — Contains the mathematical model, parameter values, and data sources underlying the model. (PDF) [file pone.0201193.s001.pdf]

The Number of Undocumented Immigrants in the United States: Estimates Based on Demographic Modeling with Data from 1990 to 2016

Supplementary Material

Mohammad M. Fazel-Zarandi* Jonathan S. Feinstein†

Edward H. Kaplan‡

Here we first explain the methods, parameter values and data sources we employ in producing our conservative estimate of 16.7 million undocumented immigrants. Then we describe the details of the simulation.

1 The Conservative Estimate

Let N_t denote the number of undocumented immigrants at time t. In our model, we set the starting date to 1990 (i.e., $t = 0$ corresponds to the beginning of

*Yale School of Management and MIT Sloan School of Management (fazel@mit.edu).

†John G. Searle Professor of Economics and Management, Yale School of Management (jonathan.feinstein@yale.edu).

‡William N. and Marie A. Beach Professor of Operations Research, Yale School of Management; Professor of Public Health, Yale School of Public Health; and Professor of Engineering, Yale School of Engineering and Applied Science (edward.kaplan@yale.edu).

1990), assuming that $N_0 = 3,500,000$, a number in agreement with the standard estimate [1].

The number of undocumented immigrants at time t is:

$$N_t = N_{t-1} + I_t - O_t, \qquad t > 0 \qquad (S1)$$

where I_t and O_t are the population inflows and outflows at time t, respectively (Table 1 summarizes notation).

Inflows: We decompose the population inflow, I_t, into (I) visa overstayers, S_t, and (II) illegal border crossers, B_t.

I. *Visa Overstayers.* Visa overstayers are non-immigrants who are admitted to the U.S. lawfully, but do not leave after the period during which they have been allowed to remain in the U.S. legally ends. DHS only started tracking visa overstayers from 2015 [2].[1] In our analysis, we focus on the 2016 visa overstayers since the 2015 number is incomplete (it only includes business and pleasure travelers). To get an estimate of the annual number of overstayers for 1990 onwards, we assume that it is proportional to the number of non-immigration visas issued by the state department, V_t, which is available for every year in our timespan [3].[2] Table 2 provides the number of visas issued for each year. Let

$$r = \frac{S_{27}}{V_{27}}, \qquad (S2)$$

[1] The visa overstay number only includes arrivals via air and sea.

[2] See [2] for the list of non-immigration visa types that can potentially lead to a visa overstay.

denote the ratio of visa overstayers to the number of individuals issued non-immigration visas for 2016 ($t = 27$ corresponds to 2016). We use as our estimate of the visa overstayers for 1990 through 2015:

$$S_t = rV_t, \qquad t \geq 1. \qquad (S3)$$

The assumption that the rate of overstays for all previous years is equal to the 2016 rate is in fact quite conservative. Let τ_j be the number of years a newly arriving undocumented immigrant in year j remains in the country. Then $\Pr\{\tau_j \geq k\}$ is the probability that a new arrival in year j is still present k years later. The total number of visa overstayers present at year t is

$$\sum_{j=1}^{t} S_j \Pr\{\tau_j \geq t - j\} = r \sum_{j=1}^{t} V_j \Pr\{\tau_j \geq t - j\}. \qquad (S4)$$

Approximately 41% of undocumented immigrants based on the current survey data approach are visa overstayers [4], which translates to a visa overstay population of 4.6 million in 2015. For formula ($S4$) to generate as many overstayers as the 4.6 million in the 11.3 million estimate, we would need to increase the visa overstay rate to $1.1 \times r$.

II. *Illegal Border Crossers.* We estimate the number of individuals who successfully cross the border in year t, B_t, using the data provided in the recent DHS report [5]. The report uses a repeated trials model [6], combined with data on apprehensions at the border, to estimate the rate of apprehension of individuals attempting to cross the Southern Border for each year from 2005 to the present (see Figure 1 for their results). In turn, these estimates

can be used to generate an estimate of B_t for each year from 2005 to the present. Let p_t be the probability (for year t) that an individual attempting to cross the border illegally is apprehended. Formula (S9) below is used by DHS to estimate p_t. Assume that an apprehended individual is returned to the original (foreign) side of the border, and that with probability d_t the individual chooses not to try again; DHS provides estimates for d_t for each year from 2005 to the present. Thus with probability $(1 - d_t)$ an individual does not give up, and tries again to cross, again facing the probability p_t of being apprehended. (We note that the deterrent effect of enforcement is implicitly controlled for, since this is one factor leading individuals to give up.) Let C_t be the total number of individuals who wish to cross the border in year t and will make at least one attempt. $C_t p_t$ individuals will be apprehended on their first attempt, and a fraction $(1 - d_t)$ of these will attempt to cross again. It follows that $C_t p_t^2 (1 - d_t)$ individuals will attempt to cross a second time and be apprehended on their second attempt. Continuing in this way, a geometric series is generated that provides a formula for the total number of apprehensions that will be made, as well as the total number of repeat apprehensions, that is, apprehensions of individuals who tried to cross and were apprehended at least once earlier in the year. Let A_t denote the total number of apprehensions (see Table 3), and $\bar{A}_t$ denote the number of repeat apprehensions. DHS [5] provides data for both of these. Applying the logic of the model:

$$A_t = C_t p_t + C_t p_t^2 (1 - d_t) + C_t p_t^3 (1 - d_t)^2 + ... \qquad (S5)$$

$$A_t = C_t \frac{p_t}{1 - p_t(1 - d_t)}, \qquad (S6)$$

$$\bar{A}_t = A_t - C_t p_t. \qquad (S7)$$

Thus,

$$\frac{\bar{A}_t}{A_t} = \frac{A_t - C_t p_t}{A_t} = 1 - \frac{C_t p_t}{C_t \frac{p_t}{1-p_t(1-d_t)}}. \qquad (S8)$$

It follows using algebra that

$$p_t = \frac{\bar{A}_t / A_t}{1 - d_t}. \qquad (S9)$$

Now let Q_t denote the number of individuals who give up without having crossed successfully:

$$Q_t = C_t p_t d_t + C_t p_t^2 (1 - d_t) d_t + C_t p_t^3 (1 - d_t)^2 d_t + ... = A_t d_t. \qquad (S10)$$

Rearranging (S6):

$$C_t = A_t \frac{1 - p_t(1 - d_t)}{p_t}. \qquad (S11)$$

The number of successful border crossers B_t is equal to the difference between the initial pool of individuals who wish to cross, C_t, and the number who give up, Q_t (all others eventually make it across successfully in this model). Thus

$$B_t = C_t - Q_t = A_t \frac{1 - p_t(1 - d_t)}{p_t} - A_t d_t. \qquad (S12)$$

Finally,

$$B_t = A_t \frac{1 - p_t}{p_t}. \qquad (S13)$$

We make a few notes about this formula. First, the probability of apprehension is assumed to be constant across attempts. This rate could decrease, if individuals learn how to escape detection over time; and it could increase due to a selection effect with individuals better able to escape detection making it through after just one or a few trials. Second, the DHS estimates of the apprehension rates in [5] are subject to uncertainty. However, their estimates are larger than those elsewhere in the literature [7, 8], thus contributing to our overall conservative estimate (underestimate) of the number of border crossers. Third, we compared the above model with models where individuals quit if they fail n times ($n > 2$). The results show that the number of border crossers in the repeated trial model is indeed lower than the crossers in these alternative models. Thus our model is again conservative in terms of the number of crossers we use in our analysis.

Most experts agree that the apprehension rate was significantly lower in earlier years and has been steadily increasing [7, 8]. Another point of data in support of this is the fact that the number of border agents has increased dramatically over the timespan of our analysis [9] (see Table 4). Moreover, the number of hours spent by border agents patrolling the immediate border area increased by more than 300% between 1992-2004, and new infrastructure (e.g., fences) and technologies (e.g., night vision equipment, sensors, and video imaging systems) were introduced during this period [10]. Thus, for our conservative estimate we assume that the apprehension rate in years

1990-2004 was equal to the average rate in years 2005-10 or 39%; this is well above the rates discussed in the literature for earlier years and thus tends to reduce our estimate of the number of undocumented immigrants since it implies a larger fraction are apprehended at the border.

Outflows: The outflow O_t is comprised of: (I) emigration, (II) mortality, (III) deportation, and (IV) adjustment of status from unauthorized to lawful.

I. *Emigration.* The emigration rate corresponds to the fraction of undocumented immigrants who leave the U.S. voluntarily. Since the propensity to emigrate decreases with the duration of stay [12], we consider duration-dependent emigration rates; thus we employ separate emigration rates for those who have spent one year or less in the U.S., 2-10 years, or longer. We base our value for the 1-year emigration rate, μ_s, on the 1-year visa overstay exit rate for 2016 ([11]; the rate for 2015 is similar), which is approximately 30% - this is the fraction of overstayers who left the country within one year from the day their visa expired. We again take a conservative stance by increasing the 1-year rate to 40%; thus among individuals who enter at time t, $0.4 \times I_t$ will leave by time $t + 1$. Note that this rate is especially an overestimate for illegal border crossers, who are widely viewed as having a lower likelihood of returning in the first year than visa overstayers [8]. For years 2-10, we draw on parameter values in the literature; these estimates fall from 0.01 to 0.04 [12-15]. For our conservative estimate calculation, we set the 2-10 year rate, μ_m, equal to 4%, the highest estimate in the literature. Published estimates of the emigration rate for individuals who have been in the country more than 10 years typically fall around 1%, thus,

we equate this rate, μ_l, to 1% per line with these estimates. Given that emigration rates depend upon time spent in the United States, the dynamic nature of our model results in different overall annual emigration rates for each year in our study, ranging from 5% to as high as 25%. These rates are significantly higher than estimates of the emigration rate for those born outside the United States found in the literature or government sources; published estimates include 1% [14 - 17], 2.4% [13], and 2.9% [12]. The main reason the emigration rates in our model greatly exceed those found in the literature is the extremely high 40% emigration rate that we assume for those who have only been in the country for one year. To further enhance the conservatism of our model, we assume that all undocumented immigrants present at the beginning of 1990 have been here for only one year.

II. *Mortality Rate.* We set the mortality rate, δ, equal to 0.7%, the age-adjusted mortality rate reported by the Center for Disease Control and Prevention [18]. Note that this is generally viewed in the literature as an overestimate [13]. To further check that this rate is an overestimate, we combined the age, gender, and country of birth distributions of undocumented immigrants reported in [17, 19] with CDC mortality rates [18] (CDC reports death rates by age, race, and Hispanic origin). The resulting mortality rate is less than 0.2%, much lower than the mortality rate we consider. Note that the mortality rate is quite small and does not have a large impact on our estimates.

III and IV. *Deportations and Adjustments.* The annual number of deportations and

adjustments (change from illegal to legal status), which we denote D_t, are taken directly from published data [13, 20, 21]. To overestimate the outflows, we include the deferred action for childhood arrivals (DACA) recipients in the annual adjustments [22]. Table 5 presents the annual number of deportations and adjustments in our timespan.

We use the following procedure to calculate our conservative estimate of the population of undocumented immigrants at each time t. Since the emigration rate depends on the duration of stay, we must keep track of entry times. If $t \leq 10$, calculating N_t is straightforward - from equation (S1) we get:

$$N_t = N_{t-1}(1 - \mu_m - \delta) + I_t(1 - \mu_s) - D_t \qquad t \leq 10. \qquad (S14)$$

If $t > 10$, however, the formula becomes more complicated, as the exit rate of the population with age greater than 10 reduces to $(1 - \mu_l - \delta)$. To incorporate this into equation (S1) let:

$$\theta_j = \begin{cases} (1-\mu_m-\delta)^{10}(1-\mu_l-\delta)^{t-10}, & j = 0 \\ (1-\mu_m-\delta)^{9}(1-\mu_l-\delta)^{t-j-9}, & 0 < j \leq t-10 \\ (1-\mu_m-\delta)^{t-j}, & j > t-10 \end{cases}$$

The number of undocumented immigrants at time $t > 10$ is then:

$$N_t = N_0\theta_0 + \sum_{j=1}^{t} \left([I_j(1-\mu_s) - D_j]\,\theta_j \right), \qquad t > 10. \qquad (S15)$$

2 The Simulation

2.1 Methodology

Our simulation is designed to evaluate the range of outcomes the model produces, thus taking into account important sources of variability. There are two main sources of uncertainty, parameter uncertainty and the inherent variability of the population conditional upon parameter values. We take both sources into account.

We address parameter uncertainty by establishing ranges for key parameters. These key parameters are:

(i) the visa overstay rate, r;

(ii) border apprehension rates for individuals attempting to cross the border illegally, $p = \{p_1, ..., p_{27}\}$ (recall $t = 27$ corresponds to the year 2016);

(iii) the voluntary emigration rate, which is set separately for illegal border crossers, μ_s^β, and visa overstays for the first year, μ_s^o; then jointly for both border crossers and visa overstays for years 2-10, μ_m; and jointly for years 10 and above, μ_l. We also establish a cohort-specific range for each annual cohort from 1991-2016 for the first-year rate for illegal border crossers, $\mu_s^\beta \equiv \{\mu_{s,1}^b, ..., \mu_{s,27}^b\}$;

(iv) the mortality rate, δ.

For each parameter we establish a uniform distribution over a set range (we will describe the parameter ranges in the next section).

To include the second source of variability, the inherently stochastic nature of the population, we impose a Poisson structure on our model. Specifically, conditional on all parameter values, which we represent by $\alpha \equiv \{r, p, \mu_s^\beta, \mu_s^o, \mu_m, \mu_l, \delta\}$, we model the overall population as the sum of Poisson variables, each of which

counts the number of people who enter at a given time and exit at a future time. Formally, let $\Lambda_{j,k}$ denote the number of arrivals at year j who are still present k years later, and $\Pr\{\tau_j^i \geq k\}$, $i \in \{o, b\}$ denote the probability that an individual undocumented immigrant in the cohort of type i (overstayer or border crosser) arriving at year j is still present k years later. Then,

$$\Lambda_{j,k} \sim \mathit{Poisson}\left(S_j(\alpha)\Pr\{\tau_j^o(\alpha) \geq k\} + B_j(\alpha)\Pr\{\tau_j^b(\alpha) \geq k\}\right), \qquad (S16)$$

[3]

and the overall population is

$$N_t = \sum_{j=0}^{t} \Lambda_{j,t-j}. \qquad (S17)$$

We assume that the Poisson variables $S_j(\alpha)$ and $B_j(\alpha)$ are mutually independent conditional on the parameters α for all time periods j, and also that $S_j(\alpha)$ ($B_j(\alpha)$) is independent of $\tau_j^o(\alpha)$ ($\tau_j^b(\alpha)$) for all j, again conditional on the parameters. The first assumption means that given the parameter values, the number of visa overstayers in any given year does not depend upon the number of border crossers in any other year. This is a reasonable assumption as possible correlations that might arise among these two arrival types are already captured in the parameters. The second assumption simply means that the duration an arriving individual remains in the country does depend upon the year of arrival, but does not depend upon the number of arrivals. Since the sum of independent Poisson variables is also Poisson, the population size N_t conditional on the parameters α is also

[3]Given that we only have yearly deportation and adjustment of status data, we adjust $\Lambda_{j,k}$ by correcting for D_j.

Poisson distributed, that is:

$$N_t(\alpha) \sim Poisson\left(\sum_{j=0}^{t}\left(S_j(\alpha)\Pr\{\tau_j^o(\alpha) \geq t-j\} + B_j(\alpha)\Pr\{\tau_j^b(\alpha) \geq t-j\}\right)\right). \qquad (S18)$$

Thus, each simulation run follows two steps: (i) a random draw of the parameter vector, which we denote by $\widetilde{\alpha}$, and a draw for the initial population of undocumented immigrants in 1990, denoted by n_0; and (ii) conditional upon $\widetilde{\alpha}$, a draw for the population at year t, $n_t(\widetilde{\alpha})$, for $t = 1, 2, ..., 27$.

2.2 Parameter Ranges

The parameters are uniformly drawn from the following ranges:

1. *Visa overstay rate*: for each simulation, the visa overstay rate is set equal to the 2016 rate multiplied by a uniform draw from the range [.5,1.5].

2. *Probability of apprehension:*

   (a) 1990 to 2004: for these earlier years we assume a uniform distribution over the range [.25,.40].

   (b) 2005 to 2015: for each year between 2005 to 2010, we use the numbers in [8] as lower bounds, the DHS numbers in [5] as the mid-points, and the DHS numbers in [5] plus the difference between the DHS numbers and the numbers in [8] as upper bounds. The apprehension probabilities are then selected at random between these lower and upper bounds. Since [8] only provides the probability of apprehension up to 2010, for 2011 to 2015, we use five year rolling averages to get the lower and upper bounds.

3. *Voluntary emigration:*

   (a) First-year rates:

      i. For visa overstayers we assume the first-year rate falls in the range [.25,.50] for each year.

      ii. For illegal border crossers, there are data indicating that first-year rates vary across cohorts [8]. To incorporate this, we assume that a voluntary emigration rate is drawn for each cohort year from a uniform distribution that is specific to that cohort's year of initial entry; the lower bound of this range is set by the numbers in [8] and the upper bound is set at 0.50.

   (b) For years 2-10 we assume a range [.01,.05].

   (c) For years 10 and above we draw a value from the range [.005,.02].

4. Mortality: this rate is drawn from the range [.005,.01].

5. To capture circular flows, we impose a negative correlation between the first-year emigration rate and the border apprehension rate for illegal border crossers; based on our own analysis for annual data from the best recent study [8] we use a correlation of -0.5. Specifically, we generate two correlated random variables, one for the probability of apprehension and the other for the first-year emigration rate of border crossers from the ranges described above.

Figures and Tables

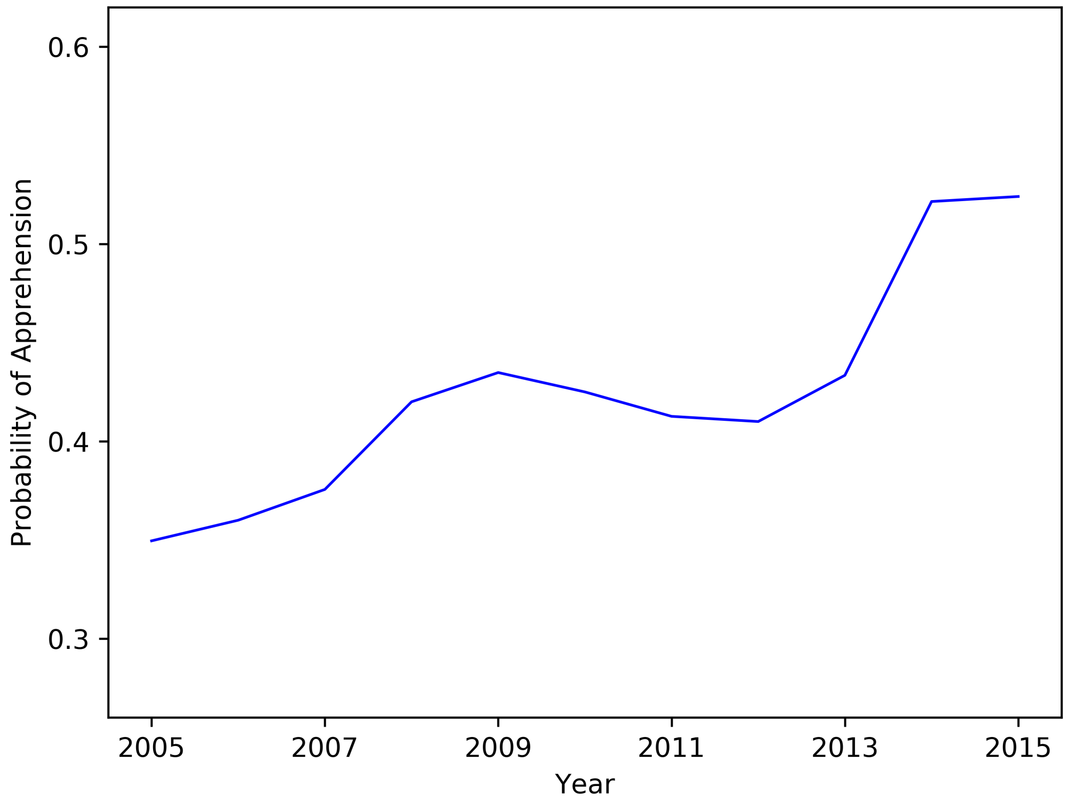

Figure 1: Southern Border Apprehension Rate 2005-2015 - Data Source: U.S. Department of Homeland Security [4]

Table 1. Notation

Parameter	Description
N_t	Number of undocumented immigrants at time t
S_t	Number of visa overstays at time t
B_t	Number of border crossers at time t
I_t	Total inflow at time t
V_t	Number of non-immigration visas at time t
C_t	Number of individuals wishing to cross the border at time t
Q_t	Number of individuals who give up without having to cross at time t
r	Overstay rate
p_t	Border apprehension rate at time t
d_t	Probability of giving up at time t
A_t	Number of border apprehensions at time t
$\bar{A}_t$	Number of repeat apprehensions at time t
μ_s	First year emigration rate
μ_m	2-10 year emigration rate
μ_l	More than 10 year emigration rate
D_t	Number of deportations and adjustments at time t
δ	Age-adjusted average mortality rate
$\Lambda_{j,k}$	number of arrivals at year j who are still present k years later
$\Pr\{\tau_j \geq k\}$	Probability that a new arrival in year j is still present k years later
O_t	Total outflow at time t

Table 2. Number of Non-Immigration Visa Issues

Year	Visas	Year	Visas
1990	4,850,670	2004	3,821,822
1991	5,026,298	2005	4,174,816
1992	4,576,644	2006	4,722,102
1993	4,418,952	2007	5,188,127
1994	4,551,906	2008	5,398,167
1995	5,106,963	2009	4,661,000
1996	5,333,575	2010	5,026,509
1997	5,162,948	2011	5,910,719
1998	5,148,774	2012	6,958,609
1999	5,164,066	2013	7,416,050
2000	5,268,821	2014	8,220,061
2001	5,241,895	2015	9,189,856
2002	4,003,965	2016	8,755,614
2003	3,642,277		

Source: U.S. Department of State [3]

Table 3. Apprehensions on the Southern Border

Year	Apprehensions	Year	Apprehensions
1990	1,049,321	2004	1,139,282
1991	1,077,876	2005	1,171,396
1992	1,145,574	2006	1,071,972
1993	1,212,886	2007	858,638
1994	979,101	2008	705,005
1995	1,271,390	2009	540,865
1996	1,507,020	2010	447,731
1997	1,368,707	2011	327,577
1998	1,516,680	2012	356,873
1999	1,537,000	2013	414,397
2000	1,643,679	2014	479,371
2001	1,235,718	2015	331,333
2002	929,809	2016	408,000
2003	905,065		

Source: U.S. Department of Homeland Security [21]

Table 4. Number of Border Agents

Year	Border Agents	Year	Border Agents
1992	3,555	2005	9,891
1993	3,444	2006	11,032
1994	3,747	2007	13,297
1995	4,388	2008	15,442
1996	5,333	2009	17,408
1997	6,315	2010	17,535
1998	7,357	2011	18,506
1999	7,706	2012	18,546
2000	8,580	2013	18,611
2001	9,147	2014	18,156
2002	9,239	2015	17,522
2003	9,840	2016	17,206
2004	9,506		

Source: U.S. Customs and Border Protection [9]

Table 5. Annual Number of Deportations and Adjustments

Year	Deportations	Adjustments	Year	Deportations	Adjustments
1990	25,369	37,883	2004	107,313	84,288
1991	28,568	35,290	2005	108,056	79,037
1992	33,921	42,925	2006	113,576	64,200
1993	34,023	44,870	2007	175,344	94,064
1994	34,921	38,392	2008	150,078	100,485
1995	35,765	41,900	2009	164,839	104,029
1996	41,426	55,428	2010	185,128	88,363
1997	58,954	54,319	2011	181,555	90,228
1998	64,797	61,448	2012	148,153	247,864
1999	65,287	60,393	2013	110,094	523,323
2000	65,279	72,621	2014	102,224	181,220
2001	71,191	176,169	2015	69,478	114,115
2002	80,836	114,927	2016	65,332	167,165
2003	101,750	119,709			

Source: U.S. Department of Homeland Security [13, 20, 21, 22]

References

[1] Krogstad JM, Passel JS. “5 Facts about Illegal Immigration in the US.” (Pew Research Center, Washington, DC, 2015).

[2] U.S. Department of Homeland Security, Fiscal Year 2016 Entry/Exit Overstay Report (DHS Report, 2017; https://www.dhs.gov/publication/entryexit-overstay-report).

[3] U.S. Department of State, Classes of Nonimmigrants Issued Visas (1990-2016; https://travel.state.gov/content/visas/en/law-and-policy/statistics/non-immigrant-visas.html).

[4] Passel, J.S. “Modes of entry for the unauthorized migrant population.” (Pew Hispanic Center, Washington, DC, 2006).

[5] Bailey J, et al. “Assessing Southern Border Security”. (Department of Homeland Security, Washington, DC, 2016; https://cis.org/sites/cis.org/files/Border-Crossing-Stats-Report.pdf).

[6] Espenshade TJ. Using INS border apprehension data to measure the flow of undocumented migrants crossing the US-Mexico frontier. International Migration Review 545-565 (1995).

[7] Wein LM, Liu Y, Motskin A. Analyzing the Homeland Security of the US-Mexico Border. Risk Analysis, 29(5), 699-713 (2009).

[8] Massey DS, Pren KA, Durand J. Why border enforcement backfired. American Journal of Sociology 121(5), 1557-1600 (2016).

[9] U.S. Customs and Border Protection, U.S. Border Patrol Fiscal Year Staffing Statistics (1992 - 2016; https://www.cbp.gov/newsroom/media-resources/stats).

[10] Roberts B, Hanson G, Cornwell D, Borger S. "An analysis of migrant smuggling costs along the southwest border" (Department of Homeland Security, Washington, DC, 2010).

[11] "Visa Overstays: a gap in the nation's border security," (U.S. House of Representatives Homeland Security Committee Hearing, Washington, DC, 2017).

[12] Van Hook J, Zhang JW. Who stays? Who goes? Selective emigration among the foreign-born. Population Research and Policy Review 30(1) 1-24 (2011).

[13] Warren R, Warren JR. Unauthorized immigration to the United States: Annual estimates and components of change, by state, 1990 to 2010. International Migration Review 47(2), 296-329 (2013).

[14] Bhaskar R, Arenas-Germosén B, Dick C. Demographic analysis 2010: Sensitivity analysis of the foreign-born migration component. US Census Bureau Population Division Working Paper 98 (2013).

[15] Mulder T. "Foreign-born emigration from the United States: 1990 to 2000". (Paper presented at the 2003 annual meetings of the Population Association of America, Minneapolis, MN, 2003).

[16] Ahmed B, Robinson JG. Estimates of emigration of the foreign-born population: 1980-1990. US Census Bureau Population Division Working Paper 9 (1994).

[17] Baker B, Rytina N. "Estimates of the unauthorized immigrant population residing in the United States: January 2012" (Department of Homeland Security, Washington, DC, 2013).

[18] Kochanek KD, Murphy SL, Xu J, Tejada-Vera B. Deaths: final data for 2014. National Vital Statistics Report, 65, 1-122 (2016).

[19] Passel JS, Cohn D. "A portrait of unauthorized immigrants in the United States." (Pew Research Center, Washington, DC, 2009).

[20] "Tracking Immigration and Customs Enforcement Removals" (Transactional Records Access Clearinghouse, Syracuse Univ., New York, 2017; http://trac.syr.edu/phptools/immigration/remove).

[21] U.S. Department of Homeland Security, Yearbook of Immigration Statistics (DHS, Office of Immigration Statistics, 2016; https://www.dhs.gov/immigration-statistics/yearbook).

[22] U.S. Citizenship and Immigration Services, Number of I-821D, Consideration of Deferred Action for Childhood Arrivals (U.S. Department of Homeland Security, Washington, DC, 2016; https://www.uscis.gov/tools/reports-studies/immigration-forms-data/data-set-form-i-821d-deferred-action-childhood-arrivals).

[23] Carriquiry A, Majmundar M, Eds, Estimating illegal entries at the U.S.-Mexico border. (National Academies Press, Washington, DC 2013).
